# Mitochondrial Ion Channels in Cancer Transformation

**DOI:** 10.3389/fonc.2015.00120

**Published:** 2015-06-04

**Authors:** Stephen M. Madamba, Kevin N. Damri, Laurent M. Dejean, Pablo M. Peixoto

**Affiliations:** ^1^Department of Natural Sciences, Baruch College, City University of New York, New York, NY, USA; ^2^City University of New York Graduate Center, New York, NY, USA; ^3^Department of Chemistry, College of Science and Mathematics, California State University Fresno, Fresno, CA, USA; ^4^Department of Basic Sciences, New York University College of Dentistry, New York, NY, USA

**Keywords:** cancer cell transformation, VDAC, MAC, PTP, TIM, TOM

## Abstract

Cancer transformation involves reprograming of mitochondrial function to avert cell death mechanisms, monopolize energy metabolism, accelerate mitotic proliferation, and promote metastasis. Mitochondrial ion channels have emerged as promising therapeutic targets because of their connection to metabolic and apoptotic functions. This mini review discusses how mitochondrial channels may be associated with cancer transformation and expands on the possible involvement of mitochondrial protein import complexes in pathophysiological process.

## Introduction

The eukaryotic merger that gave rise to mitochondria was arguably the major contributor to the origin of multicellular organisms (1, 2), afforded by the increased efficiency in cellular energy conversion. However, maintaining multicellular life required more than power. Mitochondria had to specialize in eliminating malfunctioning cells and policing unrestrained growth of once-single cells. An insurgence against the newfound multicellular way of life would be punished with a death sentence.

**Figure 1:**
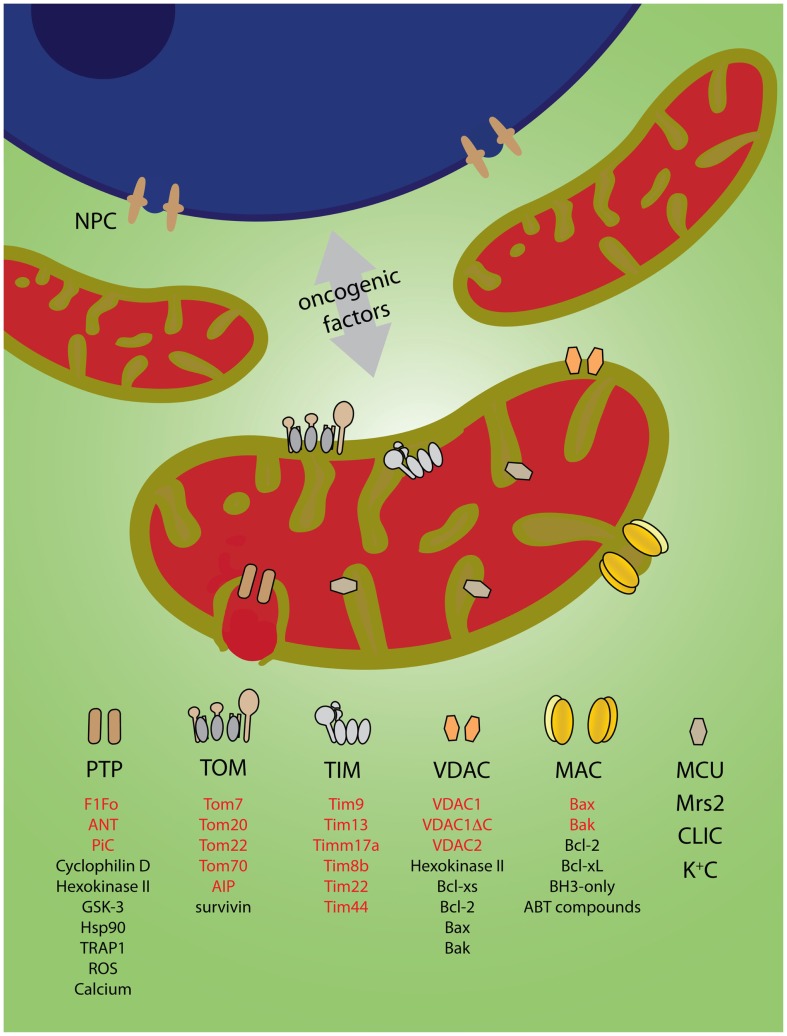
**Mitochondrial ion channels might mediate exchange of oncogenic factors with the nucleus**. Nuclear oncogenic factors either directly (via nuclear pore complexes, NPC) (12) or indirectly (via oncogenic expression) are transmitted to mitochondria to reprogram metabolism and cell death mechanisms. Channels in the outer and the inner membranes might provide the pathways for production and/or bi-directional transport of oncogenic factors. Alternatively, they might be direct targets (see text). The legend indicates channel components (red) or modulators (black) associated with cancer (see text for references). Components and regulators of TIM22 and TIM23 channels were listed in the same category. K^+^C represents the potassium channels BK_Ca_ and IK_Ca_, ATP-dependent K_ATP_, Kv1.3, two-pore TWIK-related acid-sensitive K^+^ channel-3 (TASK-3). Inspired by Odra Noel’s “Mitochondrial Dawn”.

Power, or a death sentence, is released from mitochondria through channels spanning the inner and the outer membranes. For example, mitochondria deliver ATP and other high-energy metabolites to the cell through a voltage-dependent anion channel [VDAC, reviewed in Ref. (3)], while death signals like cytochrome *c* are unleashed through the mitochondrial apoptosis-induced channel [MAC; (4)] (Figure 1). Comprehensive reviews on the structure–function relationships of ion channels in mitochondria are available elsewhere (5–11). This mini review will focus on the possible involvement of mitochondrial ion channels in cancer transformation.

If an insurgent cell is to survive, it must reprogram the power plant. Some cancer cells accomplish this task by decreasing the open probability of mitochondrial channels involved in the transport of energy metabolites (13–15) and relying on glycolytic metabolism to fuel competitive reproduction (3). One would think that the payoff for relinquishing energy conversion efficiency is that cancer cells suffer lower oxidative stress. However, oxidative stress seems to be one of the determinants of tumor cell transformation (16, 17). As discussed below and elsewhere (18), respiring mitochondria are the major sources of cytotoxic reactive oxygen species (ROS). Interestingly, exacerbated ROS emission causes the opening of pores in the inner membrane that breach the permeability barrier to solutes and the ensuing uncontrolled matrix swelling, membrane rupture, and spillage of mitochondrial proteins into the cytosol. Although the exact mechanism of permeability transition of the inner membrane is still being deciphered, cancer cells expertly avert them by equally mysterious ways.

Another feature of cancer mitochondria is that they are coated with sentinel proteins, which prevent the formation of MAC in the outer membrane, rendering the power plant oblivious to cancer transformation and unrestrained growth. Interestingly, much of our current understanding about the regulation of MAC arose from studying cancer cells, which over express the sentinel protein Bcl-2. It may also sound counterintuitive that the MAC components Bax and Bak are structurally related and belong to the Bcl-2 family of proteins. The truth is that this multifaceted family of proteins controls permeabilization of the outer membrane by either inducing or preventing it. In cancer cells, the second mode prevails and might even go beyond the outer membrane, as some anti-apoptotic members (Bcl-xL and Mcl-1) have been shown to interact with and regulate inner membrane proteins, including putative components of the permeability transition pore complex (19–21).

Induction and maintenance of tumor transformation might involve more than reprograming of metabolism and containment of apoptotic signaling. Recent studies suggest that mitochondrial dysfunction might be causally linked to the characteristic genomic instability of many human cancer cells (22–27). One could envision the involvement of additional mitochondrial channels responsible for, e.g., control of calcium, potassium and magnesium flux, organelle volume, and nucleic acid transport. The development of new tools has enabled the identification of elusive mitochondrial channels like the calcium uniporter (MCU) (28, 29), creating momentum for deeper understanding of the role of mitochondria in cancer cell transformation. Excellent reviews have been recently published on the therapeutic potential of mitochondrial ion channels in cancer (30–32). This mini review will focus on the putative role that these channels might exert on the reciprocal transmission of oncogenic factors between mitochondria and the nucleus (Figure 1).

## Metabolic Channels

### Voltage-dependent anion channel

Sometimes coined the food channel, VDAC was discovered in mitochondria almost 40 years ago (33). There is currently no definitive evidence that indicates the steady state conformation of VDAC in intact cells, but a pioneering study suggests an open state (34). When fully open, VDAC allows the exchange of ATP and ADP as well as electron transport chain substrates pyruvate, malate, succinate, and NADH across the outer mitochondrial membrane (35–41). In this state, the channel is normally slightly anion-selective but becomes cation-selective in its half-open state, possibly favoring calcium over metabolites (42). Interestingly, this cationic conductance has also been observed when VDAC is fully open (42), suggestive of a possible role in transport of larger positively charged metabolites. Although more sporadic in reconstituted systems, this behavior was observed in preparations from yeast and mammalian VDAC and might explain the overall positive net conductance of mitochondria (42–44). In mammals, three isoforms of VDAC have been discovered: VDAC1, VDAC2, and VDAC3 (45). These isoforms display similarities in both structure and function, but each has been found to play a distinct role in mitochondria-mediated apoptosis and metabolism. Little is known of the function of VDAC3, which is abundant in sperm cells. Is any particular isoform up/down regulated in different tumors? The answer seems to be yes, at least for VDAC1. Recent studies found that the *vdac1* gene is up regulated in breast, colon, liver, lung, pancreatic, and thyroid cancer tissue (46, 47). Knockdown of *vdac1* caused slowed proliferation of HeLa cells and reduced tumor size *in vivo*, suggesting a role of VDAC1 in the loss of growth control commonly observed in cancer cells (48).

Another possible role for VDAC1 may exist in tumor microenvironments, which are often hypoxic. VDAC1 with a truncated C-terminus (VDAC1-ΔC) has been identified in both cancer cell lines and patient tumor tissue samples (41). VDAC1-ΔC was present in cancer cell lines that had high-cytoplasmic levels of adenosine triphosphate and in lung cancer biopsies that showed a strong resistance to chemotherapy-induced apoptosis (49).

Voltage-dependent anion channel has become an attractive target for anticancer therapy due to its essential role in apoptosis and metabolism, processes deregulated in cancer (50). Cancer cells exhibit high levels of aerobic glycolysis, a phenomenon first observed by Otto Warburg in 1956. The glycolytic enzyme hexokinase 2 (HK2), which is overexpressed in cancer cells, is a key regulator of the Warburg effect and binds to VDAC, forming HK–VDAC complexes (51). It remains to be determined if HK2 binds to all VDAC isoforms. However, an additional link to VDAC1 and VDAC2 involvement can be inferred from their regulation by free tubulin, suggesting that it might underlie the cytotoxic effect of chemotherapeutic drugs affecting microtubule formation (15). It has also been recently suggested that the low-ATP/ADP ratio resulting from free tubulin-mediated VDAC closure is a contributing factor to the Warburg effect (12–15). VDAC2 also regulates apoptosis through its interactions with Bak, namely, the localization of Bak to the mitochondria and inhibition of Bak-mediated apoptosis (49, 52). As demonstrated in melanoma cells, overexpressing Bcl-x_S_ disrupts the VDAC2-Bak interaction by binding to VDAC2, releasing Bak, and allowing it to initiate apoptosis (53). Less is known about interaction between VDAC2 and the primarily cytosolic Bcl-2 protein Bax, but recent studies have shed some light on Bax regulation by VDAC2. Cells deficient in Bak and VDAC2, but not Bax, displayed resistance to apoptotic stimuli, suggesting a role for VDAC2 in Bax activation (54). More recently, it has been shown that VDAC2 facilitates recruitment of both Bak and Bax to the mitochondria and that VDAC2 and Bak are required for Bax-mediated apoptosis (52, 53, 55).

## Cell Death Channels

The two mitochondria-permeabilizing structures MAC and PTP are considered to be the main death channels. Of note, these two functional entities allow the release in the cytosol of mitochondrial factors such as cytochrome *c* during apoptosis and/or necrosis (56).

### Mitochondrial apoptosis-induced channel

Broadly recognized as the main site through which death signals like cytochrome *c* are released into the cytosol, MAC is a dynamic channel formed in the outer membrane by at least two pro-apoptotic members of the Bcl-2 protein family, Bak and Bax (57, 58). However, the MAC pore may not be entirely of protein nature. Some studies showed that ceramides or intermediates of ceramide biosynthesis pathway had the potential to stimulate cytochrome *c* release in a Bak- and/or Bax-dependent fashion, indicating that these lipids are potentially an integral part of MAC (59, 60). In theory, MAC function antagonizes cancer cell transformation and is, in fact, kept at bay by oncogenic gene expression. Up regulation of the Bcl-2 or Bcl-x_L_ proto-oncogenes is associated with tumor formation and more particularly with B-cell non-Hodgkin’s lymphoma (61–63). Pro-lymphocytic cell lines overexpressing Bcl-2 exhibit resistance to MAC formation and the ability to induce lymphoma in mice (44, 63–65). Paradoxically, it was shown that an over-abundance of Bcl-2 not only caused mitochondrial sequestration of BH3-only proteins, which are activators of MAC formation, but also that of the MAC core component Bax (66–68). This phenomenon seems to be a common feature of anti-apoptotic Bcl-2 family members as an over-abundance of Bcl-xL was also recently shown to induce both Bax accumulation and functional activation at the mitochondria (69, 70). This accumulation has been coined “priming for death,” and this concept is currently being used to develop new cancer therapies (66, 67). The first approach to pharmacologically trigger Bax-mediated apoptosis in primed cancer cells comes from the group of Stanley Korsmeyer. In one of these studies, a stabilized stapled BIM helix peptide was even shown to efficiently compete for binding to Bcl-2 with mitochondrial native BIM in a model of hematologic cancer (71). Another approach taken was to screen for small organic molecules, which can act as BH3 mimetics on anti-apoptotic multi-domain Bcl-2 family members. The first of these BH3 mimetic compounds to be developed was ABT-737, which binds Bcl-2, Bcl-xL, and Bcl-w (69). An orally bioavailable variant (ABT-263 or Navitoclax) was later developed (12) with promising clinical trial results but the caveat of causing thrombocytopenia (15).

ABT-199 is another recently developed drug that has shown antitumor activity *in vitro* and *in vivo* (31) and is perhaps the most promising BH3 mimetic. Unlike ABT-263 and ABT-737, ABT-199 is Bcl-2 selective and does not seem to cause thrombocytopenia (72). Its efficacy was first demonstrated in chronic lymphocytic leukemia (CLL), but its cytotoxic activity has since been reported in T-cell acute lymphoblastic leukemia (T-ALL) (73), acute myeloid leukemia (74), and chronic myeloid leukemia (CML) progenitor cells (15). In the CML study, treatment with ABT-199 in combination with the tyrosine kinase inhibitor imatinib enhanced inhibitory effects. It has also been used in combination with tamoxifen to induce apoptosis in estrogen receptor-positive breast cancer cells, in which Bcl-2 is overexpressed (70). ABT-199, alone or in combination with other anticancer agents, represents a promising strategy against growth of Bcl-2 dependent cancers.

Tumorigenesis and the accompanying resistance to apoptosis can also be initiated by down-regulation of pro-apoptotic Bax and Bak. In this case, the death program fails as MAC formation is prevented by a lack of building blocks (75). Chemotherapies in this latter case may involve the activation of Nur77 by the drug 3-Cl-AHPC; activation of Nur77 leading to the conversion of Bcl-2 into a core component of a new type of cytochrome *c* release channel (76).

### PTP

The selective induction of permeability transition in the inner membrane has obvious onco-therapeutic potential. However, most cancer mitochondria seem to be desensitized to signals that trigger permeability transition in normal mitochondria (discussed below). Interestingly, a high incidence of cancer is found in transplant patients treated with cyclosporine A, an immunosuppressant known to inhibit permeability transition of the inner membrane (77). The mechanisms of PTP inhibition in cancer cells are expertly summarized in two recent reviews by the Pinton and Bernardi groups: cancer cells down regulate PTP inducers (i.e., ROS and calcium), alter expression of chaperones that regulate PTP opening, desensitize the channel through kinase signaling pathways (GSK-3 and hexokinase II), leading to reduced mitochondrial oxidative phosphorylation regimes characterized by high ATP/ADP ratios, low-inorganic phosphate, and high-ROS levels (74, 78). Intriguingly, some of the currently proposed PTP components seem to be up regulated in cancer cells. That is the case, for example, for the c subunit of the ATP synthase (79). Other studies showed that increased levels of the c subunit induce cell death via PTP (80, 81). One possible explanation to this seemingly discrepancy is that cancer cells initially benefit from PTP function (mitochondrial dysfunction and ROS emission) to generate genome instability. As the cancer phenotype strengthens, PTP is silenced, possibly by a combination of the four mechanisms listed above, a process sometimes potentiated by an increase in mitophagy (74). It should be noted, however, that the structural composition of PTP is still a matter of debate and it might involve interactions between cyclophilin D, the adenine nucleotide transporter (ANT), the phosphate carrier (PiC), and the F1Fo ATP synthase in the inner membrane (82, 83).

## Protein Import Channels

Mitochondrial biogenesis relies on the import of over 99% of the organelle proteome from the cytoplasm. The import pathway is through water-filled channel complexes, namely, the translocase of the outer membrane (TOM), and the translocases of the inner membrane (TIM22, and TIM23) [for a comprehensive review, see Ref. (84)]. The TIM23 channel was the first electrophysiological demonstration of the link between protein import and water-filled channels in mitochondria (85, 86) followed by reports on the channel activity of TOM, the protein import complex of the outer membrane (87, 88). These two channels enable the coordinated passage of proteins across both membranes and into the matrix space.

Translocase of the outer membrane channels act as gatekeepers of protein import as they open in response to binding of precursor proteins carrying a signal peptide (89). This signal can be a cysteine motif (90), a presequence (91), or an internal targeting element (92). Most of the precursor proteins pass the outer membrane via the TOM complex. Precursors with cleavable N-terminal presequences are further sorted to the matrix or inner membrane by TIM23. Precursors of β-barrel proteins bind to small Tim chaperones in the inter membrane space and are then inserted into the outer membrane via the sorting and assembly machinery (SAM). If the precursor carries specific cysteine motifs, it will be transferred from TOM to the MIA complex for sorting into the intermembrane space. Finally, precursors of the metabolite carrier family are transferred via the Tim chaperones to the TIM22 translocase which then mediates their insertion into the inner membrane (93). A few α-helical outer membrane proteins seem to be imported independently from the TOM complex but may instead require the SAM complex (94).

A number of subunits of the protein import machineries have been found to be overexpressed in mitochondria of cancer cells, including Timm17a, Tim9, Tim13, Tim8b, Tim22, Tom20, Tom70, and Tom7 (79). While it might sound logical that enhanced biogenesis of mitochondrial proteins during cancer transformation requires up-regulation of the protein import machineries, it is surprising that, out of the list mentioned above, the only channel forming protein is Tim22. Another study found splice variants of Tim44 in oncotic thyroid carcinomas and suggests this TIM23 peripheral component might have a role in cancer transformation possibly by induction of ROS production or impairment of protein import (95). Furthermore, metabolic reprograming during tumorigenesis often requires redistribution of kinases or transcription factors to mitochondria in a process that depends on the mitochondrial import machinery (12–15).

Translocase of the outer membrane complex proteins have been shown to interact with both pro- and anti-apoptotic Bcl-2 family proteins, but their role in mitochondrial apoptosis is unclear. Tom22, essential for maintaining TOM structure, has been established as the mitochondrial receptor of Bax (96). In HeLa cells treated with the proteasome inhibitor celastrol, Tom22 was up regulated, yet Bax translocation still occurred in the absence of Tom22, Tom70, and Tom40 (97, 98). Interestingly, metaxins 1 and 2, components of the SAM complex, were found to be required for Bak activation in TNF-induced apoptosis in Bax deficient glioma cells (99).

Another TOM subunit, Tom20, might have a role in cancer transformation via interaction with the arylhydrocarbon receptor-interacting protein (AIP) (100). AIP is also associated with the inhibitor of apoptosis protein survivin, which localizes to the mitochondria and when released in response to apoptotic stimuli, prevents cell death by inhibiting caspase activation (101). Survivin is overexpressed in cancer cells and its import into the mitochondria is mediated by the AIP–Tom20 complex; this pathway may be a potential target for cancer cells (102), provided that the AIP–survivin interaction can be specifically targeted, as AIP has been shown to bind other mitochondrial pre-proteins (100).

### Small Ion Channels

Voltage-dependent anion channel is thought to allow unrestrained ion flux across the outer membrane. However, ion flow is tightly regulated across the inner membrane as to allow the vital establishment of the hydrogen proton gradient. Inner membrane ion channels control metabolic pace, emission of ROS, organelle volume, and other functions that seem to be modified in cancer cells; they are therefore natural candidates for activity modulation through cancer transformation. A non-exhaustive list of potential targets includes mitochondrial potassium channels [calcium-dependent BK_Ca_ and IK_Ca_, ATP-dependent K_ATP_, Kv1.3, two-pore TWIK-related Acid-Sensitive K^+^ channel-3 (TASK-3)], calcium uniporter MCU, magnesium channels (Mrs2), anion channels (CLIC), and channel activities that await molecular identification (mCs, AAA, ACA, and IMAC) (30–32).

## Concluding Remarks

Mitochondrial ion channels are emerging as promising onco-therapeutic targets. Compelling and mounting literature suggests that reprogramed metabolic and apoptotic signaling from mitochondria is present in cancer cells and might underlie their characteristic genome instability. The field should benefit from studies that aim at identifying oncogenic factors that are either released from mitochondria or that affect the function of these organelles. Also helpful would be the ability to manipulate specific mitochondrial functions in animal models of cancer cell transformation; and new electrophysiological and imagery techniques allowing live monitoring of mitochondrial channels in cancer cells. Cancer cell transformation, rather than being solely induced by the nucleus or the mitochondria, might involve crosstalk between these cellular compartments.

## Conflict of Interest Statement

The authors declare that the research was conducted in the absence of any commercial or financial relationships that could be construed as a potential conflict of interest.
